# Mechanical Characterization for Cellular Mechanobiology: Current Trends and Future Prospects

**DOI:** 10.3389/fbioe.2020.595978

**Published:** 2020-11-12

**Authors:** Badri Narayanan Narasimhan, Matthew S. Ting, Tarek Kollmetz, Matthew S. Horrocks, Anaïs E. Chalard, Jenny Malmström

**Affiliations:** ^1^Department of Chemical and Materials Engineering, The University of Auckland, Auckland, New Zealand; ^2^MacDiarmid Institute for Advanced Materials and Nanotechnology, Wellington, New Zealand

**Keywords:** biointerfaces, mechanical properties, mechanotransduction, cell-substrate interactions, atomic force microscopy, traction force microscopy, Brillouin microscopy, magnetic tweezers

## Abstract

Accurate mechanical characterization of adherent cells and their substrates is important for understanding the influence of mechanical properties on cells themselves. Recent mechanobiology studies outline the importance of mechanical parameters, such as stress relaxation and strain stiffening on the behavior of cells. Numerous techniques exist for probing mechanical properties and it is vital to understand the benefits of each technique and how they relate to each other. This mini review aims to guide the reader through the toolbox of mechanical characterization techniques by presenting well-established and emerging methods currently used to assess mechanical properties of substrates and cells.

## Introduction

When engineering mechanical cues to study cell mechanotransduction, it is important to take into account how the cells sense the mechanical properties. Material mechanical properties ought to be measured at timescales and frequencies relevant to the properties sensed by adhering cells (Cameron et al., 2014; Charrier et al., 2018). When cells interact with a substrate, they can apply forces parallel and perpendicular to the surface (Janmey et al., 2020). This implies that the method used for assessing cellular forces may affect the results in mechanotransduction studies. One more factor to consider is the depth into a material that cells can sense the mechanical properties. Modeling, and studies on thin gels, suggest that cells sense mechanical properties in the range of their radius (Sen et al., 2009; Buxboim et al., 2010; He et al., 2014). The range within which cells can sense mechanical properties may be material-dependent though, and it has been shown that strain stiffening fibrous networks enable long-range force sensing of up to hundreds of microns (Leiphart et al., 2019). It is therefore relevant to consider the sensing depth of surface and bulk mechanical characterization techniques, respectively. How cells sense their environment is also cell-type dependent and, while many excellent model studies have been performed in 2D, cell sensing is likely to be different in three-dimensional (3D) culture environments (Doyle and Yamada, 2016).

**FIGURE 1:**
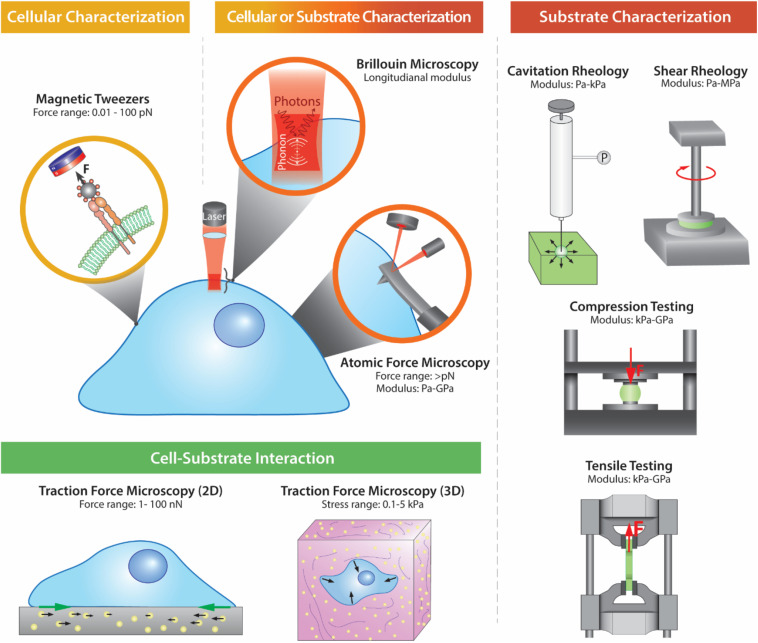
Schematic illustrations of the different methods to characterize the mechanical properties of substrates and/or cells.

Many model substrates have been adopted to study cellular mechanotransduction. In 1980, silicone rubber was first used as a deformable elastic substrate for cell culture, which revealed that cells constantly exert forces on substrates (Harris et al., 1980). Building on this work, gels gained interest as model substrates due to their tunable viscoelastic properties and close resemblance, mechanically, to the extracellular matrix (ECM) (Tibbitt and Anseth, 2009; Chaudhuri, 2017). However, thin films and liquids have also been used as substrates (Yang et al., 2014; Kong et al., 2018). To precisely determine the mechanical cues that substrates display to cells is crucial for biological studies. It is also important to assess the changes in the mechanical properties of cells in response to various biophysical and biochemical cues. Therefore, this review describes and contrasts the most commonly used techniques to determine the mechanical properties of materials and cells (Figure 1 and Table 1).

**TABLE 1:** Considerations and relevant examples for the various characterization techniques to assess mechanical properties at different length scales, ranging from cm to nm.

Method	Considerations	Examples
Global scale (cm-mm) Shear rheology	– Sample’s contact ensured by applying a normal force (high normal forces = higher values of estimated modulus) – Slippery gels: use of crosshatched or sandpaper geometry	– Normal force-controlled rheology of polyethylene glycol composite hydrogels (Randriantsilefisoa et al., 2020) – Geometry with sandpaper to probe silk-alginate hydrogel (Ziv et al., 2014)
Compression testing	– Preload applied to the samples to ensure contact (not always relevant for fragile materials) – Difference between confined and unconfined testing	– Biphasic theory combined with confined compression tests to assess collagen hydrogels mechanical properties (Busby et al., 2013)
Tensile testing	– Use of dog-bone shape samples to avoid breakage and sandpaper to avoid slipping at the clamps	– Use of digital image correlation to assess mechanical properties of slippery samples (Subhash et al., 2011)
Cavitation rheology	– Complicated measurements when probing size scales similar to sample defects – Different types of cavitation (needle or laser-based) that operate at different strain regimes	– Probing stiffness gradients in supramolecular hydrogels (Thomson et al., 2020) – Comparison of elastic properties of gels using bulk and cavitation rheology (Bentz et al., 2018)
Brillouin microscopy	– Biophysical interpretation of the measured signal still causes debate	– Reply to “Water content, not stiffness, dominates Brillouin spectroscopy measurements” (Scarcelli and Yun, 2018)
Traction force microscopy	– Use of a reference image of cells and beads in a stressed state first, and then measurement of the beads’ displacement after removal of the cells in a relaxed state	– Reference free TFM methods (Balaban et al., 2001; Bergert et al., 2016)
Probe-based techniques	– Tip selection based on the expected stiffness of the material	– Nanoindentation vs. AFM indentation with tip selection considerations (Qian and Zhao, 2018) – Nanomechanical mapping of soft substrates (Garcia, 2020)
Magnetic tweezers Local scale (nm)	– Magnetic field orientation can be used to produce different types of forces and torques – Difficult to use synergistically with other techniques	– A guide to magnetic tweezers (Sarkar and Rybenkov, 2016) – Detecting bound proteins on DNA using hybrid magnetic and optical tweezers (van Loenhout et al., 2013)

## Assessment of the Global Mechanical Properties

The mechanical properties of substrates such as gels used in mechanotransduction studies are usually characterized globally by rheology, tensile, and compression testing.

Shear rheology can be used to monitor the gelation process, or to measure the elastic and viscous properties of gels (denoted by storage and loss modulus) by applying small amplitude oscillatory shear forces (Zuidema et al., 2014). Time-dependent aspects of viscoelasticity are quantified by relaxation time and relaxation modulus (Fitzgerald et al., 2015; Chaudhuri et al., 2016). Strain stiffening materials are characterized by monitoring the elastic modulus as a function of increasing stress. It is important to note the significance of the geometries used for probing the sample. For instance, a recent study showed that a crosshatched geometry revealed the strain stiffening property of agarose, while a parallel plate geometry obscured it due to the wall slippage effect (Bertula et al., 2019). The main limitation of rheology to characterize gels is that the spatial variation in moduli arising from sample heterogeneities cannot be probed.

Compression and tensile testing are also techniques commonly used to probe the global mechanical properties, especially for biomaterials (Xiao et al., 2013; Vedadghavami et al., 2017). The elastic, or Young’s modulus, of the sample is generally obtained from the slope of the stress-strain curve, but the viscoelastic properties can also be accessed through this kind of characterization (Mirahmadi et al., 2013; Bosnjak et al., 2020). Variations in the testing protocols can, however, influence the results (Patel et al., 2019) and difficulties can also arise in tensile testing from the breakage or slipping of the sample at the clamps (Oyen, 2014).

Several of the aforementioned mechanical parameters can be probed using different techniques, and it is important to understand how the parameters are measured. For example, tensile testing measures the elastic properties (Young’s modulus) by applying tensile forces, while in rheology shear forces are applied (storage modulus).

## Assessment of the Local Mechanical Properties

The local mechanical properties are relevant for measuring what properties cells sense at the surface of a material. The resolution of the techniques described below enable the mechanical properties of cells to be probed in real time due to their specificity and sensitivity.

### Probe-Based Techniques

The atomic force microscope (AFM) belongs to a family of instruments known as scanning probe microscopes. These instruments are commonly used for obtaining high resolution, 3D images of a sample surface using a tip which scans across an area of interest whilst measuring the interactions with the surface. Force-indentation is one major application of the AFM, measuring the interaction forces between the probe tip (sharp or colloidal) and a sample surface (Yablon, 2013). A force-indentation curve is obtained by measuring the displacement of the tip at the end of a cantilever, monitored by a laser and photodiode while controlling the force of the indentation. AFM has been extended for studying biological samples and their processes (Alsteens et al., 2017; Krieg et al., 2019).

The main advantage AFM force-indentation has over traditional nanoindentation is the ability to apply a lower range of well-defined forces (pico-Newtons to nano-Newtons) by changing the cantilever spring constant or tip, which is particularly useful for hydrogel or soft biological samples. Sub-pico-Newton forces have also been achievable by improving tip stability (King et al., 2009; Churnside and Perkins, 2014). Some challenges associated with AFM force-indentation are that the indentation parameters and cantilever stiffness must be appropriately selected and accurately calibrated to model the data to mechanical information. A variety of methods exist for accurate cantilever calibration, the most common being the thermal noise calibration method (Hutter and Bechhoefer, 1993). Laser Doppler vibrometry has also been used to achieve more accurate calibrations (Ohler, 2007; Gates and Pratt, 2012). Radmacher and colleagues have developed a standardized nanomechanical procedure for AFM on polyacrylamide hydrogels and MDCK-C11 cells showing improvement in consistency and reproducibility across eleven research groups in Europe (Schillers et al., 2017).

The tip and cantilever used for indentation are important for modeling the force-indentation curves obtained from AFM. Tips are usually made from stiff materials (silicon, silicon nitride or, in the case of colloids, glass or polystyrene) to be suitable for modeling, and to avoid deforming or damaging the tip when performing indentations. AFM tips can generally be classified as blunt or sharp tips. Blunt or rounded tips have a larger contact area and can achieve higher forces for the same indentation depth compared to a sharp tip (Qian and Zhao, 2018). Single cells have also been used as probes to achieve pico-Newton forces (Helenius et al., 2008; Li et al., 2019). Several different models are available to model force-indentation curves, each associated with its own assumptions and limitations (Lin and Horkay, 2008). Force-indentation data is preferably fitted in the first few hundreds of nanometers of the approach curve. The elastic modulus is usually obtained by fitting Hertz model with a rounded tip; although, sharp tips are commonly used for their availability and simplicity (Sirghi et al., 2008; MacKay and Kumar, 2012). It has also been demonstrated that the shear modulus can be obtained from Hertz modeling of force-indentation curves (Berry et al., 2020).

AFM viscoelastic measurements such as stress relaxation and creep (Yango et al., 2016; Efremov et al., 2017), strain stiffening (Van Helvert and Friedl, 2016) and nano-rheology (Li et al., 2014) are also possible. Oscillatory AFM has been used to determine the complex shear modulus of cells (Alcaraz et al., 2003). The storage stiffness, loss stiffness, and loss tangent of polyacrylamide hydrogels have similarly been determined using a magnetically driven cantilever (Nalam et al., 2015). Nano-rheology is still a relatively new technique, but future improvements of nano-rheological modeling in AFM software are expected to enhance the field significantly.

Viscoelastic modeling can be performed using a variety of models such as the Maxwell and the Voigt-Kelvin models (Cheng et al., 2005; Fischer-Cripps, 2011) or Ting’s viscoelastic model (Ting, 1964), which has been only recently applied to AFM force-indentation curves (Brückner et al., 2017; Efremov et al., 2017, 2019b). For a recent review that highlights the various models and properties obtainable from soft biological samples with AFM, the reader is directed to Efremov et al. (2019a).

#### Nanomechanical Mapping

AFM modes to map mechanical properties on the nanoscale, while imaging, have also been developed. Generally, nanomechanical mapping modes are capable of mapping the elastic and loss moduli in a range between 1 kPa and 100 GPa across an entire imaging area (100 nm^2^ to 100 μm^2^) (Morales-Rivas et al., 2015; Viji Babu et al., 2019). This technique offers high spatial resolution and can be operated in air, liquid and vacuum. The main benefit of this method compared to force-indentation is the imaging feature and the ability to overlay mechanical data and topography. Different AFM manufacturers provide integrated modes for nanomechanical mapping.

Generally, in nanomechanical mapping, individual force curves are recorded as the tip is oscillating in tapping mode and scanning the surface. The maximum force exerted on the sample is kept constant by the feedback loop, which ensures the protection of the tip and the sample. The resulting force curves are converted into force indentation curves, which can subsequently be analyzed with regards to mechanical properties such as modulus, adhesion, dissipation or deformation. The spring constant of the cantilever and the tip radius have to be calibrated prior to quantitative measurements. The operating range of this technique ranges from very soft samples (low kPa) to hard materials (100 GPa) and has been demonstrated on amyloid nanofibrils (Sweers et al., 2011), proteoglycan mimetic nanoparticles (Hedayati and Kipper, 2018), and microvilli on living cells (Schillers et al., 2016). A protocol to analyze the mechanical properties of very soft materials (cells) can be found in reference (Hu et al., 2020).

We believe the use of fast, spatially resolved, mechanical, and topographical analysis will be increasingly valuable for mechanotransduction studies in the future.

### Traction Force Microscopy

While the mechanical properties of substrates are important to characterize, an understanding of the forces that cells can exert on their surroundings is central for our understanding of mechanotransduction. Traction force microscopy (TFM) is a technique used to assess forces that cells apply to the ECM or substrate they grow on/in, by monitoring the displacement of fluorescent beads embedded in the substrate (Hur et al., 2020). Some studies also measure these forces through the deflection of elastic micropillar arrays onto which cells are adhering (Schoen et al., 2010), or use the cell-induced alignment of collagen fibers to deduce the applied traction forces (Shakiba et al., 2020).

Different kinds of TFM exist according to the number of dimensions considered, and the accuracy of the method actually increases with the number of dimensions involved in the calculations. So far, most of the materials used as substrates are hydrogels that exhibit linearly elastic and isotropic properties such as silicon or polyacrylamide hydrogels (Polacheck and Chen, 2016). However, these materials do not always reflect the behavior of native tissues. Some studies develop TFM methods that take into account the non-linear behaviors of matrices, such as fibrillar networks like collagen, or strain-stiffening materials (Steinwachs et al., 2016; Song et al., 2020). Integrating these parameters often complicates the modeling, but significantly improves the applicability of the results. TFM-based methods are also developed to focus on cell-cell and intracellular traction forces (Maruthamuthu et al., 2011), which is particularly relevant for cells of tissues with cell junctions, such as the endothelium. Recently, traction force microscopy was combined with microarray-based techniques to enable high throughput screening of cell generated forces (Kaylan et al., 2017).

## Emerging Techniques

Recent research has propagated the development of modern characterization techniques, all with unique advantages and limitations. Some of the most promising emerging techniques for mechanical characterization are summarized in this section.

Brillouin microscopy was originally demonstrated on cells over a decade ago (Scarcelli and Yun, 2008) and has since garnered attention within the biological and medical research fields for its novel approach to non-destructive viscoelastic measurements of biological samples. The method involves interacting the intrinsic acoustic waves of a material with probe laser pulses and interpreting how the light is scattered – which is heavily linked to the material’s viscoelastic properties (Gusev and Ruello, 2018). Recent works have demonstrated a submicrometric spatial resolution (Caponi et al., 2020). Brillouin microscopy is also largely non-destructive while still possessing resolution capabilities for cellular characterization (μm scale) (Prevedel et al., 2019). Since its pioneering application in 2005 (Koski and Yarger, 2005), Brillouin microscopy has been utilized in tissue-level sensing (Mathieu et al., 2011; Margueritat et al., 2019) and subcellular-level characterization (Steelman et al., 2015; Bevilacqua et al., 2019). However, interpretation of results requires specific knowledge of the refractive index and material density (Prevedel et al., 2019) which is difficult to experimentally attain (Liu et al., 2016), and the weakness of the measured signal causes extended scanning and data acquisition times (Ballmann et al., 2019). Scrutinous evaluation of Brillouin output is also required, especially in biological matter, due to the spatiotemporal inhomogeneity causing differences in the material’s intrinsic acoustic wave behavior (Wu et al., 2018; Ballmann et al., 2019).

Microfluidic devices constitute an efficient and relatively high-throughput method to characterize the mechanical properties of cells. For instance, micropipette aspiration on microfluidic chips using giant vesicles as cell models have been developed to access cell membrane mechanics (Elias et al., 2020). Other techniques use flow field analysis to study the deformations applied by fluids to cells to deduce their viscoelastic properties (Guillou et al., 2016). The influence of fluid flows on cells has also been combined with computational models to determine the elastic modulus of cells (Song et al., 2012) and such flows have also been demonstrated to impact cell behavior and maturation (Song et al., 2013; Shemesh et al., 2015).

Optical tweezers (OTs) is a single-molecule technique initially designed in 1986 (Ashkin et al., 1986). OTs involve the precise manipulation of beads (0.1–100 pN) with molecules tethered to them by trapping them with strongly focused light. In the context of mechanobiology, OTs have been used to assess ligand-receptor bond properties using ligand-functionalized trapped particles (Jiang et al., 2003), to explore forces on the structures at the surface of the cell involved in mechanotransduction (Choquet et al., 1997), to assess forces on structures within the cell (Welte et al., 1998), and to investigate the mechanical properties of the cell (Tan et al., 2012). OTs are, however, accompanied by drawbacks such as photo-entrapment of impurity particles, local heating and photodamage (Peterman et al., 2003). Some of these challenges are solved by magnetic tweezers (MTs). This single-molecule technique is relevant for biological applications such as single-molecule force measurements and cellular micromanipulation (Basoli et al., 2018). It operates by utilizing superparamagnetic beads (0.1–100 μm) as a force transducer for a tethered single large molecule by applying a magnetic field (Xin et al., 2017). Advantages of MT include its cost-effectiveness and ease of implementation, superior to other single-molecule techniques (Kemmerich et al., 2016). Furthermore, MTs can generate forces inside closed opaque bodies, such as living cells, which is not the case with other non-invasive methods (Timonen and Grzybowski, 2017). MTs have been used extensively to generate tension on cell adhesion molecules, and then observing the cellular response to reveal mechanotransduction pathways(Guilluy et al., 2014; Marjoram et al., 2016). The main challenge in MT development has been their subpar resolution that is limited by relying on camera-based measurements of magnetic beads (De Vlaminck and Dekker, 2012). However, high spatiotemporal resolution magnetic tweezers (Å scale) are being implemented with help from more sophisticated cameras and computer hardware (Huhle et al., 2015; Ostrofet et al., 2020). There are also difficulties working with particles susceptible to magnetic fields. Furthermore, the requirement that bulky magnets must be situated close to the sample complicates the combination of MTs with other applications (Sarkar and Rybenkov, 2016).

Concerning substrates, a relatively new and inexpensive technique to probe local mechanical properties is cavitation rheology (Zimberlin et al., 2007). Cavitation rheology quantifies the elastic properties by growing a bubble in a gel or fluid and monitoring the pressure dynamics (Hashemnejad and Kundu, 2017). The resolution of cavitation rheology can go as low as 1 μm and requires only small sample volumes (Barney et al., 2020). One drawback of this method is that it is destructive to the sample. Questions also remain around relating gel fracture to cavitation and the influence of local network structures on cavitation bubble growth (Barney et al., 2020).

## Conclusion and Outlooks

One of the fundamental challenging aspects of characterizing substrates is the relevance of a particular technique in capturing the properties of substrates experienced by the cell. Each technique probes the mechanical properties in a different way, and it is necessary to compare the parameters obtained using different techniques. Rheology or compression tests, for instance, can provide the elastic and loss modulus of a material, but only as a mean value for the whole material, whereas probe-based techniques such as AFM assess the local mechanical properties at the microscopic scale, which can differ from the macroscopic one. A recent study showed that the bulk elastic modulus obtained for stiffer gels using rheology and AFM correlate well but showed marked differences for softer gels (Megone et al., 2018). There has also been a recent trend in using simple and inexpensive techniques for probing mechanical properties. For instance, using fluorescent bead density as a readout for stiffness gradients in gels instead of using expensive techniques like AFM (Barber-Pérez et al., 2020). Recent technology also allows assessment of mechanical properties of materials with extremely precise force (resolution down to 10 nN claimed by the manufacturer) through micro-scale compression testing, particularly for soft biomaterials (Kerscher et al., 2017).

Emerging techniques are now focusing on probing these properties even more precisely and in-depth. Cell-substrate interactions are also highly relevant to assess mechanotransduction and techniques such as traction force microscopy and magnetic tweezers can be applied to survey such interactions. Forces in the order of pico-Newtons, applied by integrins on ligands, have also been assessed by DNA mechanotechnology (Brockman et al., 2018; Glazier et al., 2019; Ma et al., 2019). However, significant challenges regarding the integrity of the DNA probes in cell culture conditions and the type of receptor-ligand interactions that can be probed remain (Ma and Salaita, 2019; Yasunaga et al., 2019).

Many of the existing tools for measuring the mechanical properties of cells are limited in terms of throughput. To mitigate this issue, high throughput based methods have been explored to separate large populations of cells based on mechanical properties using microfluidics and shear stress (Otto et al., 2015). A combination of smart design of microfluidic geometries and powerful computational approaches such as microparticle image velocimetry and machine learning promise to further progress the field of mechanobiology (Song et al., 2010; Guillou et al., 2016).

The influence of substrate mechanical properties on processes such as nascent protein deposition has been explored in recent studies by using metabolic labeling techniques (Loebel et al., 2019, 2020). It would be interesting to combine such labeling with 3D traction force microscopy in order to directly visualize the effects of cell traction forces on nascent protein deposition.

Going forward, standard mechanical characterization techniques combined with newer, more accurate and more local assessments of the mechanical properties, will enable the development of more complex engineered matrices. Such materials with spatiotemporally-controlled mechanical properties will further the understanding of how cells interact with their environment *in vitro*, and indirectly *in vivo*. This is important to fully use the potential of stem cells in tissue engineering and stem cell therapy.

## Author Contributions

All authors listed have made a substantial, direct and intellectual contribution to the work, and approved it for publication.

## Conflict of Interest

The authors declare that the research was conducted in the absence of any commercial or financial relationships that could be construed as a potential conflict of interest.
